# 

**Published:** 1894-05-05

**Authors:** 


### CONTENTS.

**Modern Surgery** . . . . . . . . . . . . 89
**Around the Hospitals** . . . . . . . . . . 90
**On Modern Progress in Ophthalmic Medicine and Surgery.** By ROBERT BRUDENELL CARTER, F.R.C.S. . . . 91
**The Educational Treatment of Idiots, Imbeciles, and Feeble-minded Children.** By G. E. SHUTTLEWORTH, B.A., M.D. . . . . . . . . . . 92
**Studies in Applied Sociology.** VIII.—The Cure of Stammering 93
**Modern Views of Malaria.** II. . . . . . . . . 94
**Annotations.**—Alcohol as a Physiological Peacemaker—Diet and Stupidity—The Advancement of Science by Dining . . . 95
**Notes and News** . . . . . . . . . . . 96
**Scraps and Gleanings** . . . . . . . . . . 108
**Medical Progress and Hospital Clinics**—
On the Causes of Hæmorrhage During Pregnancy . . . . 97
Gout in Hospitals . . . . . . . . . 98
MEDICAL PROGRESS—Renal Affections . . . . . . 99
PROGRESS IN SURGERY—The Surgical Treatment of Pulmonary Cavities and Localised Areas of Tuberculous Lungs . . . 102
NEW DRUGS AND PRAPARATIONS . . . . . . . 103
NEW APPLIANCES AND THINGS MEDICAL . . . . . . 104
**The Institutional Workshop**—
HOSPITAL FINANCE. VII.—The Defects of Medical Assurance 105
HOSPITAL FESTIVALS, MEETINGS, &c.—Metropolitan and National Nursing Association . . . . . . . . 107
**The Book World of Medicine and Science** . . . . 109

### EXTRA SUPPLEMENT.—THE NURSING MIRROR.

**News from the Nursing World.**—"The Hospital" Convalescent Fund—Missing Names—The Pennefather Memorial—Help for the Helpless—Improvements at Wigan—Fa thful Service—A Successful Bazaar—The Berwick Ladies—Good Work at Taunton—Queen's Nurses in Wales—Loving Service Appreciated—Kindly Co-operation—The Ceylon Nurses' Association—Strength Used, Not Wasted—Short Items . xli
**On General Nursing.** X.—Pneumonia (*continued*) . . xliii
**The Memorial to Mrs. Wardroper** . . . . . xliv
**Hospital Treatment—Appointments** . . . . . xlvi
**Presentations—Our Paris Letter** . . . . . xlvi
**Women's Work.**—Women as Horticulturists . . . . xlvii
**The Ethics of Nursing.**—III. . . . . . . . xlvii
**Everybody's Opinion** . . . . . . . . . xlviii
**The Muses' Looking-Glass—Minor Appointments** . xlix
**Notes and Queries** . . . . . . . . . xlix
**Stockton and Thornaby Nursing Association** . . l
**Death in Our Ranks—For Reading to the Sick** . . l

